# The Credible Role of Curcumin in Oxidative Stress-Mediated Mitochondrial Dysfunction in Mammals

**DOI:** 10.3390/biom12101405

**Published:** 2022-10-01

**Authors:** Muthuswamy Sathyabhama, Loganathan Chandramani Priya Dharshini, Adhimoolam Karthikeyan, Senthil Kalaiselvi, Taesun Min

**Affiliations:** 1 Department of Biotechnology, PSG College of Arts & Science, Coimbatore 641014, Tamil Nadu, India; 2 Subtropical Horticulture Research Institute, Jeju National University, Jeju 63243, Korea; 3 Department of Biochemistry, Biotechnology and Bioinformatics, Avinashilingam Institute for Home Science and Higher Education for Women, Coimbatore 641043, Tamil Nadu, India; 4 Department of Animal Biotechnology, Jeju National Animal Research Center (JIA) and Sustainable Agriculture Research Institute (SARI), Jeju National University, Jeju 63243, Korea

**Keywords:** curcumin, mammals, mitochondrial dysfunction, oxidative stress, antioxidant, anti-inflammatory, anticancer, antimicrobial

## Abstract

Oxidative stress and mitochondrial dysfunction are associated with the pathogenesis of several human diseases. The excessive generation of reactive oxygen species (ROS) and/or lack of adequate antioxidant defenses causes DNA mutations in mitochondria, damages the mitochondrial respiratory chain, and alters membrane permeability and mitochondrial defense mechanisms. All these alterations are linked to the development of numerous diseases. Curcumin, an active ingredient of turmeric plant rhizomes, exhibits numerous biological activities (i.e., antioxidant, anti-inflammatory, anticancer, and antimicrobial). In recent years, many researchers have shown evidence that curcumin has the ability to reduce the oxidative stress- and mitochondrial dysfunction-associated diseases. In this review, we discuss curcumin’s antioxidant mechanism and significance in oxidative stress reduction and suppression of mitochondrial dysfunction in mammals. We also discuss the research gaps and give our opinion on how curcumin research in mammals should proceed moving forward.

## 1. Introduction

Mitochondria are membrane-bound organelles involved in oxidative phosphorylation, which is solely responsible for synthesizing phospholipids, heme, and adenosine-5′-triphosphate (ATP). It has a considerable role in calcium proportion, induction of apoptosis, and cell senescence [1]. Mitochondria possess their own genetic material and are introspective of bacterial origin. The nuclear genome encodes a few respiratory proteins of mitochondria and some mitochondrial tRNA encoded by mitochondrial genes [2]. The biogenesis of mitochondria requires both nuclear and mitochondrial genomes to express respiratory chain proteins [3]. Defects in mitochondrial expression may lead to numerous dysfunctions including diabetes mellitus, leigh syndrome, and Leber’s hereditary optic neuropathy [4,5]. Mitochondrial dysfunction is defined by the deficit of its efficacy in minimizing high-energy molecules, namely ATP required for metabolism in the body. It is related to aging and is also important in many chronic diseases [6]. Mitochondrial dysfunction is often maternally inherited by offspring from mothers. It may also arise due to inadequate mitochondrial numbers in a cell or even mutations in the mitochondrial DNA. The symptom is chronic fatigue, and the disorder cannot be cured completely but can be sustained with supportive treatments [7].

ATP synthesis occurs in mitochondria as it is the site for oxidative metabolism. Glucose, a main energy source for cellular metabolism, which is absorbed from the food consumed by the mitochondria to produce energy moieties known as ATP. Glucose is converted to ATP through the following cycles: glycolysis, Kreb’s cycle, and oxidative phosphorylation. In the glycolysis pathway, glucose is converted into pyruvate, generating two ATP molecules which are a very low energy source. Simultaneously, pyruvate is transformed to acetyl coenzyme A (acetyl Co-A), which on entering Kreb’s cycle generates 36–38 ATP molecules, enabling the oxidation of NADH and FADH_2_. These two molecules are utilized by the mitochondrial respiratory chain, protein complexes catalyze ADP phosphorylation to ATP by generating a high proton gradient across the inner mitochondrial membrane. These ATP molecules are further utilized by the other cells of our body to perform various metabolic activities [8,9].

Oxidative stress, a disproportion between the generation and over-accumulation of reactive oxygen species (ROS) and antioxidants [10]. ROS are free oxidative radicals and no radical derivatives of oxygen present in the body that can readily combine with other biomolecules resulting in the formation of toxic substances. These toxic substances formed from ROS can eventually lead to cell death. ROS includes peroxides, superoxides, hydroxyl radicals, ozone, and nascent oxygen molecules [11]. Under favorable and regulated conditions, these ROS act as signaling molecules in the cell organelles. When produced in higher amounts, it becomes a toxicant, damaging most of the biomolecules because of its ability to oxidize nucleic acids, lipids, and proteins [12]. ROS generally forms highly stable molecules, but after being oxidized with the free compounds available in the body, it becomes a toxic substance in the organelle [13].

In eukaryotes, mitochondria are a rich source of free radicals [14]. Oxidative damage induced by free radicals can potentially damage the mitochondrial DNA (mtDNA), which affects its function within the cells and contributes to redox signaling for the rest of the cell organelle [15]. ROS is generated in the mitochondrial respiratory chain with the help of enzymes and matrix proteins of the TCA cycle, and the first report of mitochondrial ROS was in 1966 [16]. To mitigate and regulate oxidative stress caused in the body, mitochondrial ROS scavenging systems come into the act, where hydrogen peroxide arises from superoxide radical dismutation with superoxide dismutase are detoxified with catalysts such as glutathione peroxidase breaking down hydrogen peroxide into water [17]. Mitochondria possesses sodium dismutase (SOD), MnSOD, which explains mitochondrial superoxide production [18]. Deregulated ROS and oxidative levels in mitochondria lead to various pathogenesis in the human body, causing mitochondrial dysfunction. Mitochondrial ROS pool has a major role in disease pathophysiology and therapeutic purpose [19]. The dysfunction in mitochondria is characterized by higher oxidative stress, nitric oxide (NO) synthesis, and declined ATP production/oxygen consumption [20]. Antioxidants scavenge ROS by donating their electron to prevalent ROS and neutralizing it. This scavenging activity of antioxidants decreases or delays the capacity of damage to macromolecules [21]. Antioxidants such as glutathione and uric acid are found during the body’s normal metabolism. At the same time, some others are found in our diet. Other lighter antioxidants, namely vitamin E, C, and β-carotene, must be supplied through diet [22,23,24].

In the view of increasing disease conditions in humans, many medicinal and dietary plants grabbed the attraction of researchers as therapeutic agents. One such plant compound is curcumin. It is a polyphenol compound present in rhizomes of turmeric plants (*Curcuma* spp.). Curcumin is beneficial as it has antioxidant, anti-inflammatory, antimicrobial, antimutagenic, and anticancer activities [25,26,27,28]. Another important mechanism is the antioxidant action of curcumin against oxidative stress [29] by increasing the effect of superoxide dismutase, glutathione and catalase, which reduces mitochondrial oxidative stress [30]. The three redox sites of curcumin can undergo oxidation and hydrogen abstraction, resulting in the formation of phenoxy radicals and stabilization across the keto-enol structure. The administration of curcumin can inactivate stress-sensitive kinases by scavenging free radicals, which could significantly prevent cell damage [31]. Consumption of natural antioxidants such as curcumin will be useful to control oxidative stress caused in our body. It combats various forms of free radicals, namely ROS and reactive nitrogen species (RNS); it modifies glutathione, SOD, and catalase activity to neutralize ROS/RNS and inhibits certain enzymes generated by ROS, such as cyclooxygenase/lipoxygenase and xanthine hydrogenase/oxidase [32,33]. It is an effective scavenger of peroxy radical as it is a lipophilic compound similar to vitamin E, and is considered a chain-breaking antioxidant [34]. Curcumin increases the ROS levels in cancer cells, which leads to apoptosis using caspase enzymes and Cytochrome C release from mitochondria. It has a potent role in regulating cancer cell proliferation and apoptosis activation in different types of cancer [35]. Curcumin can act as an antioxidant that can potentially neutralize free radical ROS in mitochondria and other cellular parts, proving a dynamic approach to controlling mitochondrial oxidative stress. In this review, we discuss curcumin’s antioxidant mechanism and the positive effect rendered by curcumin on oxidative stress-mediated mitochondrial dysfunction in mammals. In addition, we evaluate the study gaps and offer our thoughts on future curcumin research in mammals

## 2. Antioxidant Mechanism of Curcumin

**Figure 1:**
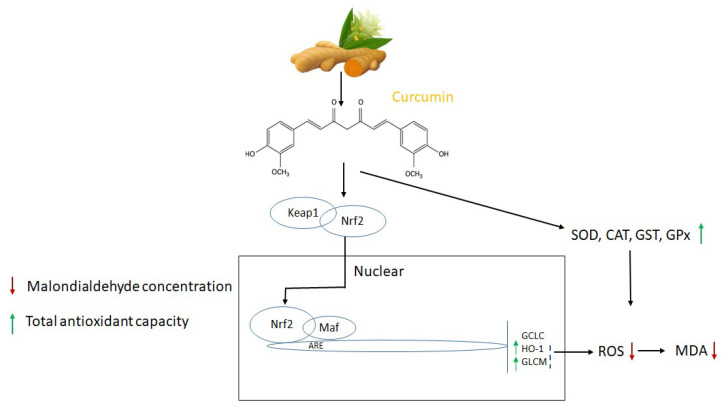
Molecular targets and antioxidant mechanism of curcumin. The activities of superoxide dismutases (SOD), catalase (CAT), reactive oxygen species (ROS), malondialdehyde (MDA), glutathione S-transferases (GST), and glutathione peroxidase (GPx) adapted from [39,40,44,45].

Curcumin possesses numerous pharmacological activities (antiangiogenic, antioxidant, antiviral, anti-inflammatory, antileukemic, immunostimulant, decarboxylase inhibitor, COX-2 inhibitor, metal chelator); therefore, it has been utilized experimentally and therapeutically in humans and animals. Of these, the antioxidant is remarkable, and the existing research outcome showed that it is an effective antioxidant that reduces the negative effects of oxidative stress [36,37]. Curcumin has the ability to prevent oxidative degradation of lipids, hemoglobin, and DNA by potentially chelating heavy metals or controlling the activity of many enzymes [38]. Curcumin’s antioxidant mechanism is summarized in Figure 1. Regarding curcumin’s antioxidant properties, research revealed that its two phenolic sites enable it to scavenge a number of free radicals directly. It has been shown to be effective against ROS and RNS production in the microenvironment. Additionally, curcumin lowers low-density lipoprotein (LDL) and prevents DNA and protein damage.

On an enzyme level, curcumin reduces the production of ROS by the enzymes (i.e., lipoxygenase/cyclooxygenase and xanthine dehydrogenase/oxidase). It increases the enzyme activity SOD and POD, which are known as the first line of defense against oxygen-free radicals [39,40]. The topical application of curcumin is well-known to obstruct TPA-induced H_2_O_2_ production in the epidermis [41]. In a study, curcumin alleviated the decrease in cardiac antioxidant enzymes (SOD and CAT) and glutathione levels (glutathione-S-transferase) in diabetic rats [42]. After inducing Al^3+^ metal ion into *Drosophila melanogaster,* Oyetayo et al. (2020) [43] found that antioxidants, including catalase, glutathione-S-transferase, and glutathione, decreased whereas H_2_O_2_ and NO, which are related to free radical precursors were increased. Notably, oxidative damage induced by the Al^3+^ ion was reduced by curcumin in a concentration-based dosage.

## 3. The Physiological and Molecular role of Curcumin in Reducing Oxidative Stress and Preventing Mitochondrial Dysfunction

**Figure 2:**
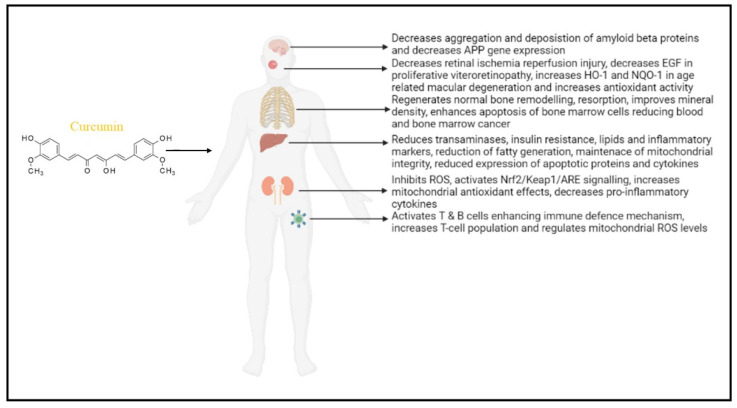
Role of curcumin in reducing mitochondrial dysfunction in various organs: in the brain—Alzheimer’s disease; eye—retinal infection; skeletal system; liver function; kidney disease, and lymphocyte regulation. Curcumin mainly regulates ROS levels and maintains the antioxidant system for proper regulation of mitochondrial function. (APP—amyloid precursor protein; EGF—epidermal growth factor; HO-1—Heme oxygenase 1; NQO1—NAD(P)H quinone oxidoreductase 1; Nrf2—nuclear factor erythroid 2-related factor 2; Keap1—Kelch-like ECH-associated protein 1; ARE—antioxidant response element.).

Elevated ROS levels and oxidative stress are associated with mitochondrial dysfunction, affecting various cellular activities. Curcumin has phenolic and β-diketone functional groups, which helps it to be an antioxidant and free radical scavenger. It enhances the activities of SOD, CAT, and GP_X_. Curcumin’s ability to penetrate mitochondria protects against oxidative damage and prevents mitochondrial dysfunction. Curcumin’s significance in reducing mitochondrial dysfunction in various organs depicted in Figure 2.

### 3.1. The Effect of Curcumin in Neurodegenerative Diseases

Neurodegenerative disease (ND) is characterized by losing an accessible population of anatomical and physiological related neurons due to other metabolic or toxic disorders. It is classified based on clinical features, anatomic distribution, and molecular abnormality [46,47]. Despite various symptoms and pathology in neurological disorders, recent evidence shows that mitochondria damage plays a considerable role in the progression of neurodegenerative disease [48]. In ND patients, combining quercetin and curcumin enhanced neuro and mitochondrial-protective effects against the side effects of oxaliplatin. It declines lipid peroxidation levels, protein carbonyl content, and simultaneous oxidative stress in mitochondria. It also increases electron transport chain complex enzymes and alters enzymatic and non-enzymatic antioxidants [49]. Curcumin can protect the central nervous system in NDs. It prevents dysfunction in mitochondria and suppresses neuronal death by targeting various pathways, including ROS, intrinsic/extrinsic apoptosis pathway, inflammatory mediators, and microglial cells. It also reduces the loss of neurons and neurotoxic compounds. Curcumin also protects the central nervous system (CNS) against ischemia-induced mitochondrial dysfunction and the onset of NDs [50].

Alzheimer’s disease (AD) is the most common ND characterized by oxidative damage in mitochondria leading to degeneration. These damages enhance oxidative stress causing mitochondrial dysfunction in AD [51]. Studies revealed that higher levels of oxidative damage to biomolecules were observed in AD patient’s brains. β-amyloid is an essential protein in AD which misfolds and forms aggregates due to oxidative stress. The β-amyloid protein in mitochondria interacts with alcohol dehydrogenase inhibiting cytochrome c oxidase [52,53,54]. In human brain tissues and transgenic mice from AD, it was observed that there are direct interactions between ROS and amyloid plaques [55]. The β-amyloid protein impairs mitochondrial dynamics, declines mitochondrial biogenesis, synaptic activity, and improves mitochondrial function. Curcumin enhances mitochondrial fusion activity, biogenesis, and synaptic proteins. It also increases cell function and viability in SHSY5Y cells [56].

### 3.2. The Effect of Curcumin in Liver Function (Alcoholic Fatty Liver and Obesity)

Fatty liver is the condition in which over-accumulation of fats occurs in hepatocytes [57]. It is the earliest change observed in the pathology of non-alcoholic fatty liver disease (NAFLD) and alcoholic fatty liver disease (AFLD) [58]. NAFLD may be due to the insulin resistance linked with metabolic risk factors [59]. AFLD is due to excessive consumption of alcohol [49]. Both cases lead to steatohepatitis followed by fibrosis, cirrhosis, hepatocellular carcinoma (HCC), and simultaneously death [60]. In patients with NAFLD, insulin resistance is likely to occur along with mitochondrial dysfunction, which plays a significant role in the progression of NAFLD to non-alcoholic steatohepatitis (NASH) [61,62]. High free fatty acids (HFFAs) induced mitochondrial impairment and oxidative stress in primary hepatocytes. Treatment with curcumin inhibited ROS production, ATP depletion, and lipoapoptosis, contributing to the cell’s survival and regaining the membrane potential of mitochondria. Curcumin also increases the copy number of mitochondrial DNA (mtDNA) along with higher levels of transcription factors, namely peroxisome proliferator-activated receptor-γ coactivator 1α (PGC1α), nuclear respiratory factor 1 (NRF1), and mitochondrial transcription factor A (Tfam) regulating mitochondrial biogenesis [63,64]. Curcumin reverses the role of HFFA-induced enhancement of PGC-1α levels, thereby upregulating mitochondrial biogenesis in fatty liver patients [65]. In an experiment with high-fat diet (HFD)-induced obese mice (OM), mitochondria from the liver were isolated, showing high oxidative stress. On treatment with curcumin, it was observed that there was increased oxygen consumption and decreased lipid and protein oxidation levels in isolated mitochondria compared with untreated obese mice [66,67]. Curcumin reduces body weight, decreasing body fat in HFD-induced OM mice [68].

### 3.3. The Effect of Curcumin in Renal Function

The kidney filters out waste products and withholds other proteins and other components. In the case of damage, these products will drain into urine from the blood. The filters were slowly shut down and lost their ability to filter [69]. Chronic kidney disease (CKD) and chronic renal failure (CRF) are the disease condition that prevails with the abnormality of kidney failure for more than three months period [70]. CKD occurs when a person is diagnosed with anemia, hypertension, breathing shortness, kidney function alterations, itches, cramps, damage to the glomerular capillary tubes, cognitive changes, and occurrence of peripheral odema due to accumulation of sodium [71]. CKD patients are more prone to muscle atrophy with the decline in physical exercise, which contributes to hazardous situations and decreases the quality of life. However, no preventive measures or treatment for muscle atrophy have been devised so far [72,73]. Oxidative stress-mediated mitochondrial dysfunction contributes widely to muscle atrophy. Mitochondria has a considerable role in generating energy as ATP for metabolism in muscle and ROS production [74]. Curcumin, a promising candidate, prevents CKD-induced muscle atrophy by improving mitochondrial dysfunction, biogenesis, and oxidative metabolism of mitochondria. Thereby, it decreases the level of oxidative stress in CKD patients with muscle atrophy [75,76]. Furthermore, pre-treatment with curcumin will protect the renal function by the early decline in changes of CKD-induced mitochondrial biogenesis, oxidative damage, and dynamics [77]. Patients with CRF are inevitably prone to cardiovascular diseases linked with higher oxidative stress associated with mitochondrial dysfunction. Renal failure was observed after nephrectomy in Wistar rats, followed by alteration in cardiac functions. Treatment with curcumin ameliorates the cardiac problem linked with the decline in ROS production, a decrease in oxidative stress markers, and enhanced antioxidant activities [78].

Various documentation alleviates the role of curcumin protective activities against several disease models mediating the mechanism of protecting mitochondrial function and maintaining its integrity [79]. A study using curcumin enhanced the antioxidant enzyme activity levels and the oxidative stress decline. It prevented the capacity of respiration in mitochondrial isolation in nephrectomy, followed by heart failure induced by cardiac reperfusion rats [79,80]. Gentamicin (GM)-induced renal injury is closely linked with mitochondrial dysfunction in proximal convoluted tubules [81]. An experiment conducted in both in vitro cell culture of tubular cells and in vivo studies in rat kidneys exposed to GM brings about the disruption of mitochondrial membrane potential, decline in production of ATP, release of cytochrome c oxidase, apoptosis, and decrease in antioxidant status [82,83]. Treatment with curcumin rendered a positive effect against GM-induced renal injury by protecting antioxidants and modifying the inflammatory response by nuclear factor—κB [84,85]. In 5/6 nephrectomized rats (a rat model with one kidney removed totally and the other 2/3 of the other kidney removed a week later), curcumin reduced the expression of NFκB signaling, and increased NRF2 translocation, enhanced antioxidant enzymes, and decreased inflammation [86,87].

### 3.4. Effect of Curcumin in Eyes (Retina)

Curcumin has been shown to have negative effects on RPE cells at concentrations indicated as effective in the treatment of tumor cells and reducing the death of retinal neurons (∼10 µm). It is recommended that the function of retina must be closely monitored while taking curcumin as a concurrent therapy for cancer or in the treatment of visual problems. Increased oxidative stress has a role in the etiology of a number of binding retinal disorders (i.e., diabetic retinopathy, retinitis pigmentosa, and age-related macular degeneration) [88]. Antioxidants may reduce the likelihood of developing age-related macular degeneration [89]. Curcumin has been proposed to have potential benefits in reducing the progress of diabetic retinopathy, age-related macular degeneration, and retinitis pigmentosa based on results acquired in animal models of retinopathies and cultured retinal cells [90]. In the retina of hyperglycemic rats, dietary curcumin reduced oxidative alterations and suppressed the increased activity of interleukin-1β, tumor necrosis factor (TNF)-α, and vascular endothelial growth factor (VEGF) [91]. In a rat model of light-induced retinal degeneration, dietary curcumin also prevented the activation of inflammatory genes and protected retinal cells from oxidative damage and simultaneous leading to cell death [92]. Curcumin reduced staurosporine-induced retinal ganglion cell and murine amacrine cell death, decreased neuronal apoptosis, and microvessel degeneration in an experimental ischemia-reperfusion retinal injury model in rats with retinitis pigmentosa. It has also been found that curcumin causes human retinal endothelial cells to undergo apoptosis [93].

It is unclear how curcumin affects retinal pigment epithelial (RPE) cells. RPE cells defend the outer retina from photo-oxidative stress during the digestion of shed photoreceptor outer segments transporting oxidized lipids, avoiding retinal edema and neovascularization [94]. Age-related macular degeneration’s pathogenesis heavily depends on RPE cell malfunction and degeneration [95]. The wet form is recognized by choroidal neovascularization and subretinal edema brought on by outer retinal hypoxia, whereas the dry form is distinguished by the presence of lipofuscin inside the RPE and drusen under the RPE, both of which include photoreceptor-derived oxidized lipids. VEGF is the main hypoxia-induced angiogenic agent that triggers the development of retinal neovascularization and edema. RPE cells are a significant source of VEGF in the retina. Basic fibroblast growth factor (BFGF), a growth factor in addition to VEGF, regulates retinal neovascular diseases such as diabetic retinopathy and the most common kind of age-dependent macular retinopathy. In hyperactive retinopathies and choroidal neovascularization, HGF-induced cell scattering is a necessary step for RPE cell migration and proliferation. Recent research has shown that curcumin reduces RPE cell viability by inducing caspase activation [96]. Researchers evaluated the toxicity of curcumin in human RPE cells as well as curcumin’s effects on the production and release of angiogenic factors from the cells.

The retina is a component in the CNS that comprises the posterior region of the optic globe, and is directly in touch with the vitreous fluid. It is made up of several cell types, of which two kinds of photoreceptors are described in detail: the rods, which are intense at the retina’s periphery, function in scotopic bright conditions (< 0.1 lux, night vision) and are especially susceptible to darkness; while the cones are intense in the macula lutea, and are more sensitive to fine shape and light, being able to distinguish colors and working in photopic light conditions (> 10 lux). The retina also has bipolar cells, amacrine cells, horizontal cells, Muller cells, and retinal pigment epithelial cells in addition to photoreceptors (RPEC). The RPE is a retinal layer that is responsible for numerous important tasks, including the digesting of damaged photoreceptor outer segments (POS) and maintenance of retinal structure. Munia et al. studied the effects of resveratrol, lutein, and curcumin on human retinal epithelial cells, finding that pre-treatment with these nutraceuticals shielded these cells from demise following oxidative stress. The retina is a continual oxidative stress target. It is identified as being mitochondrial-rich and with capillaries that are constantly affected by photons of light. This explains why the majority of retinal disease developments include an oxidative stress equilibrium with high amount of ROS and low levels of antioxidant scavengers.

Furthermore, it is important to note that the retina has a high amount of polyunsaturated fatty acids (PUFA) and, as previously stated, a higher oxygen and glucose intake in contrast to other tissues; these properties make the retina very susceptible to oxidative stress. The RGCS and photoreceptors, in particular, are vulnerable to oxidative stress damage. ROS imbalance is well known to be involved in several retinal diseases, including uveitis, age-related macular degeneration (AMD), diabetic retinopathy (DR), central serous chorioretinopathy (CSC), macular edema (ME), and from uncommon etiologies, retinal ischemia-reperfusion injury (RIRI), retinal and choroidal tumors, proliferative vitreoretinopathy (PVR), hereditary tapeto-retinal degenerations, and retinal and choroidal tumors.

Curcumin substantially decreased retinal vascular leakage in a diabetic retinopathy (DR) animal model. (1) Curcumin is an antioxidant that inhibits free radical formation [97]. (2) Curcumin boosts the mRNA expression of antioxidant enzymes such as SOD and catalase by lowering oxidative DNA damage and controlling nitrosative DNA damage [98]. (3) Curcumin activates a mitochondrial pathway by controlling the respiratory activity of mitochondrial complexes I, II, III, and V while simultaneously activating Nrf2 [99]. (4) Curcumin, a dual inhibitor of dual inhibitor of arachidonic acid, can increase antioxidant capacity in the retina of diabetic rats as well as hypoglycemic and preventive anti-inflammatory activity by lowering levels of proinflammatory cytokines such as IL-1, tumor necrosis factor-alpha, VEGF and 5-hydroxyeicosatetraenoic acid [100]. (5) Curcumin inhibits the migration of retinal human endothelial cells and functions as an antiangiogenic drug by reducing stromal cell-derived factor 1 alpha.

### 3.5. Effect of Curcumin on the Skeletal System

Mitochondrial dysfunction can cause damage to the osteoblasts, the cells that help the bone formation and mineral absorption, ultimately leading to bone diseases [34]. Mitochondrial oxidative stress can cause an imbalance between osteoblast and osteoclast’s functions within the skeletal bones [101]. Thus, this imbalance will lead to bone-related disorders such as osteoporosis, osteoarthritis, and the demineralization of bones. Consumption of curcumin can improve the proliferation and differentiation of these cells resulting in normal bone remodeling, resorption, and improving the mineral density of the skeletal system [102]. However, some experiments demonstrate that curcumin administered at appropriate dosages can regulate the reactive oxygen species levels in normal proportions within a cell, beyond which it could have some negative impacts and inhibiting effects on the cell organelles [103]. Apart from oxidative stress and mitochondrial dysfunction, curcumin is also known to cause apoptosis of bone marrow cells, reducing the risk of blood and bone marrow cancer.

### 3.6. Effect of Curcumin on the Lymphatic System

It is known that curcumin can activate T and B cells of the lymphatic system providing an efficient immune defense mechanism to the body with the help of using natural compounds [104]. Curcumin can increase the T cell population over the tumor site of the affected individuals or organisms in the circulating lymphatic system, proving that the compound can be used to fight against malignant cancers known so far [105]. It inhibits the tube formation in rat lymphatic endothelial cells, thereby exhibiting anti-lymphangiogenic effects [106]. It can regulate the mitochondrial ROS level of lymphocytes, thus aiding in the lymphatic system’s normal metabolism [107]. 

### 3.7. Effect of Curcumin on Psychiatric Disorders

Depression is a chronic psychological condition that reduces one’s quality of life and increases one’s risk to death. It is a complex disorder with multifactorial etiologies that includes genetic and environmental influences [108]. Curcumin has been widely used as an antidepressant in modulating neurotransmitters (increases in noradrenaline, dopamine, serotonin and decreases in monoamine oxidase enzymes), improving mitochondrial protection, decreasing levels of oxidative markers and nitric oxide, increasing antioxidant enzyme activity, restoring HPA axis dysfunction and enhancing auto-immuno inflammatory action [109]. Table 1 and Table 2 illustrate the cause of mitochondrial dysfunction and the role of curcumin against mitochondrial dysfunction in various organs, respectively.

## 4. Conclusions and Future Perspectives

In comparison to other plant-derived compounds, curcumin has garnered considerable attention for its therapeutic value over the years. In this review, according to the content mentioned, numerous research has demonstrated curcumin’s potent ability to reduce oxidative stress and prevent mitochondrial dysfunction. However, they are limited to in vitro and in vivo studies, and no detailed data for clinical trials about the long-term effects and precise mechanisms of curcumin on oxidative stress in humans were available. As a result, we know little about the potential risks of curcumin and its modified formulations in human. Therefore, a greater number of clinical studies must be conducted to comprehend the possible advantages of curcumin and its modified formulations and related risks in humans. Curcumin’s mechanism of action is complex and linked to multiple signaling pathways. Its targeting mechanisms are not well understood. Therefore, the precise molecular targets and regulatory mechanisms of curcumin require further investigation. It is hoped that further studies on curcumin will provide novel insights to curcumin’s role in reducing oxidative stress and preventing mitochondrial dysfunction.

## Figures and Tables

**Table 1:** The molecular causes of mitochondrial dysfunction in various organs in the body.

Organ	Causes of Mitochondrial Dysfunction	Affected Genes/Proteins	Anticipated Disease State	Reference
Brain	Excessive accumulation of calcium in the mitochondrial matrix Opening of mitochondrial permeability transition pore Release of cytochrome C leading to activation of apoptosis Dysfunction in fission and fusion activities in mitochondria	Cyclophilin D (Cyp D) Cytochrome C (Cyt C) Mitofusin (Mfn) Dynamin-related protein1 (Drp1) Optic atrophy mitochondrial protein (OPA)	Traumatic brain injury (TBI) Alzheimer’s disease Parkinson’s disease Huntington’s disease Ischemic stroke	[110]
Liver	Inner mitochondrial lesions Dynamic alterations in mitochondria Lower levels if respiratory chain complex enzymes Inability to synthesize ATP	Nuclear factor- κB (NF- κB) I kappa B-kinase (IKK-α,β,γ) Stimulation of Interferon genes (STING) TANK binding kinase 1 (TBK1) Interferon regulatory factors (IRF_3_, IRF_7_)	Non-alcoholic fatty liver disease Alcoholic fatty liver disease Drug-associated fatty liver disease Hepatitis B Hepatitis C	[111]
Lungs	Increased concentration of iron mitochondria Abnormal metabolic activity due to excessive mtROS production Decrease in mitochondrial number and function	Mammalian target of rapamycin (mTOR) Peroxisome proliferator- activated receptor gamma coactivator 1-alpha (PGC-1α) Angiotensin converting enzyme 2 (ACE2) Tumor necrosis factor-α (TNF-α) Interleukin-6 (IL-6) Matrix metalloproteinase 2 (MMP2) Transforming growth factor-β (TGF-β)	Cystic fibrosis Asthma Pneumonia Tuberculosis Lung cancer Chronic obstructive pulmonary disease (COPD)	[112]
Eye	Defects in mitochondrial respiratory chain subunit complex I enzymes Deletion of mitochondrial DNA Fragments in mitochondrial network Loss of membrane potential Unproper arrangement cristae structure of optic nerve mitochondria	OPA 1 and 3 Thymidine phosphoryase (TYMP) Adenine nucleoside translocator 1 (ANT1) Twinkle mtDNA helicase (PEO1) DNA polymerase subunit gamma 1 (PLOG1)	Dominant optic atrophy (DOA) Leber Hereditary optic neuropathy (LHOA) Chronic progressive external ophthalmoplegia (CPEO) Pigmentary retinopathy	[113]
Skeletal system	Lower levels of mitochondrial enzyme production Decreased ATP production Decline in mitochondrial density Lower protein levels in ATP synthase subunit β Insulin resistance	Cytochrome C oxidase (COX) Forkhead box class-I (FoxO1) PGC-1α NADH dehydrogenase subunit IV (NADH) Protein kinase B (AKT)	Aging Cancer cachexia Disuse-induced muscle atrophy	[114,115]
Lymphatic system	Decreased ATP production Lower levels of mitochondrial respiratory chain complex enzymes	Adenylate kinase 2 (AK2) Tafazzin, Phospholipid-Lysophospholipid Transacylase (TAZ)	Severe combined immune deficiency disease (SCID)	[116]

**Table 2:** The role of curcumin in alleviating mitochondrial dysfunction in different organs.

Disease	Action of Curcumin	Effects of Curcumin	Animal Model/Cell Type	Reference
Chronic kidney disease (CKD)-induced muscle atrophy	Inhibition of GSK-3β activity	Improves muscle function Higher ATP levels Suppressing mitochondrial membrane potential Decreases mitochondrial oxidative stress and increases antioxidant levels	C57BL/6 mice	[117]
Neurodegenerative disease	Inhibits GFAP, vimentin and Prdx6 upregulation	Suppresses oxidative stress-induced inflammation Alleviates apoptosis Suppresses mitochondrial fragmentation	Human glioblastoma cell line -A172 Human astrocytes cell line derived from spinal cord- HA-sp	[118]
Insulin resistance in non-alcoholic fatty liver disease	Inhibits lipoapoptosis, ROS generation and ATP depletion	Lowers high free fatty acid-induced synthesizes of phosphoenol pyruvate carboxykinase (PEPCK) and glucose-6-phosphate Contributes cell survival Restores mitochondrial membrane potential	Hepatocytes	[66]
Hyperglycemia	Inhibits increased oxygen consumption and decreased nitric oxide levels	Decreased state 3 oxygen consumption rate Declines the levels of thiobarbituaric acid-reactive substances	Female and male heterozygote non-diabetic db/+ mice	[119]
Heart failure	Acts as an adjuvant therapy	Inhibits mitochondrial impairment Alleviates oxidative stress Decreases mitochondrial membrane potential collapse	Male wistar rats	[120]
Alzheimer’s disease	Protects β-amyloid protein	Enhances mitochondrial fusion activity Decreases fission machinery Increased biogenesis and synaptic proteins	SHSY5Y cells	[56]
Acute kidney injury	Suppresses NF-κB activation in reducing inflammation and stimulates NRF2/HO-1 signaling reduced mitochondrial dysfunction	Decline in the level of mitochondrial ROS Reduced mitochondrial fragmentation level Enhanced TCA cycle, mitochondrial biogenesis	Human renal proximal tubular epithelial cell (TEC) line—HK2	[121]

## Data Availability

No new data were created or analyzed in this study. Data sharing is not applicable to this article.
